# From Sheltered in Place to Thriving in Place: Pandemic Places of Aging

**DOI:** 10.1093/geront/gnad087

**Published:** 2023-07-07

**Authors:** Valerie Chang Greer, Andy Hong, Sarah L Canham, James Agutter, Ivis Garcia, Jess M Van Natter, Natalie Caylor

**Affiliations:** College of Architecture and Planning, University of Utah, Salt Lake City, Utah, USA; College of Architecture and Planning, University of Utah, Salt Lake City, Utah, USA; Healthy Aging and Resilient Places Lab, College of Architecture and Planning, University of Utah, Salt Lake City, Utah, USA; College of Architecture and Planning, University of Utah, Salt Lake City, Utah, USA; College of Social Work, University of Utah, Salt Lake City, Utah, USA; College of Architecture and Planning, University of Utah, Salt Lake City, Utah, USA; Department of Architecture and Urban Planning, Texas A&M University, College Station, Texas, USA; Healthy Aging and Resilient Places Lab, College of Architecture and Planning, University of Utah, Salt Lake City, Utah, USA; Medical College of Georgia, Augusta, Georgia, USA

**Keywords:** Aging in place, Covid-19, Older people, Photovoice

## Abstract

**Background and Objectives:**

Response to the coronavirus disease 2019 pandemic required rapid changes to physical, social, and technological environments. There is a need to understand how independent-living older adults are adapting to pandemic-borne transformations of place and how environmental factors may shape experiences of aging well in the context of a public health emergency response.

**Research Design and Methods:**

We conducted a photovoice study to examine the characteristics associated with aging in place. Our study investigated how independent-living older adults characterized aging in a “right” place approximately 1 year after the onset of the pandemic.

**Results:**

Six themes categorized into 2 groups capture how older adults describe a “right” place to age. The first category, “places as enactors of identity and belonging,” describes the significance of places contributing to intimate relationships, social connections, and a sense of personal continuity. The second category, “places as facilitators of activities and values,” recognizes environments that promote health, hobbies, goals, and belief systems. Participants reported modifying their daily living environments with increased use of technology and more time outdoors.

**Discussion and Implications:**

Our findings emphasize older adults’ active engagement with place and strategies used to maintain healthy aging despite public health restrictions. The results also identify place-based characteristics that may help overcome stressful circumstances from older adults’ perspectives. These findings inform pathways to pursue to facilitate resiliency for aging in place.

Aging in place (AIP) reflects personal and policy goals, with definitions varying from “never leaving one’s home” to “staying out of a nursing home” to “having choices” about where to age (Forsyth & Molinsky, 2021). Early definitions of AIP focus on remaining in one’s residence, whereas contemporary definitions have expanded to more holistic views, such as “One’s journey to maintain independence in one’s place of residence as well as to participate in one’s community” (Rogers et al., 2020, p. 1). Evolving definitions reflect a shift from associating “place” with a residential domain to considering the affective ties to a place that are essential in community domains (Coleman & Kearns, 2015). Concepts of AIP have also developed to emphasize agency and engagement that older people have in the places they live (Buffel et al., 2013; Wiles et al., 2012). Policy-based definitions of AIP currently include “meeting the desire and ability of people, through the provision of appropriate services and assistance, to remain living relatively independently in the community in his or her current home or an appropriate level of housing” (World Health Organization, 2004, p. 9) and the “ability to live in one’s own home and community safely, independently, and comfortably, regardless of age, income, or ability level” (US Center for Disease Control & Prevention, p. 1). These definitions reflect functional and emotional dynamics between people and place, and provide context for the aim of this paper, which is to explore how older adults characterize AIP amidst rapid environmental change brought about by the coronavirus disease 2019 (COVID-19) pandemic.

Two theoretical frameworks are foundational to conceptualizing aging and place in environmental gerontology; the ecological and the social-constructivist theories (Forsyth & Molinsky, 2021; Iecovich, 2014). Lawton and Nahemow’s (1973) ecological theory of aging focuses on person–environment interaction where the objective characteristics of environments, such as mobility, proximity to amenities, and security, contribute to the “fit” between people and places. The ecological theory offers the Model of Competence-Press to evaluate the positive, neutral, or negative interplay between the demands of a place and the physical and functional capabilities of people as they age (Lawton & Nahemow, 1973). Golant (2011, 2015) advances a holistic consideration of barriers that older adults might experience and acknowledges that the “right” place to age for many older people may not be in their own home; Golant holds that “rightness” of place should be matched to a person’s unique needs and vulnerabilities. In articulating the Model of Residential Normalcy, Golant distinguishes between concepts of residential comfort (where places match individuals’ unique capacities and lifestyles) and residential mastery (where people feel empowered and in control of activities and surroundings). This model underscores the entwinement of physical and emotional relationships between people and their homes and communities throughout the aging process, and connects the relationship between the well-being of older people and the places where they live.

Complementary to an ecological framework, a social-constructivist view emphasizes how places become meaningful to people over time. Rowles (1983) articulates three dimensions of emotional attachment to place—physical, social, and autobiographical insideness—which integrates places and events with a sense of self through constructing meaning. Here, changing relationships between older people and communities are understood through the significant dimensions of places across the life span (Coleman & Kearns, 2015; Gardner, 2011; Peace et al., 2011; Wiles et al., 2012). Expanding on this social-constructivist view, Gardner (2011) recognizes various types of places based on layers of emotional attachment and meaning in the aging process. Gardner identifies “first” places as home environments, “second” places as those associated with work and contribution, and “third” places as public spaces or environments that enable people to connect to broader communities (Oldenburg & Brissett, 1982). Although definitions and meanings associated with types of places vary based on individuals, this framework provides researchers an objective way to recognize and analyze emotional attachment to place. Wiles (2005) further conceptualizes this social-constructivist view by describing places as processes, arguing that places are in constant change, as are people’s ideas and associations with place, creating a simultaneous material/physical and social/symbolic dynamic between aging and place.

Chaudhury and Oswald (2019) connect the ecological and social-constructivist theories through an Integrative Framework that distinguishes between processes of building agency and belonging through relationships with place. The processes of building agency focus on tangible, goal-directed behavior in the physical and social environment (objective), though measures of “fit” vary depending on the person and the place. In addition, processes that contribute to a sense of belonging relate to affective ties and cognitive evaluations of place (subjective), which are a constantly constructed. Chaudhury and Oswald’s Integrative Framework provides a model for understanding how agency creates pathways to reinforcing autonomy while belonging creates pathways to reinforcing a sense of identity. It also reflects the fluid and dynamic nature of relationships between people and place in the aging process. The multidimensional aspect of place is significant in both ecological and social-constructivist theories (Low & Altman, 1992; Rowe & Kahn, 1997). Attachment to place exists within the larger context of life-course events, resulting in emotional qualities suffusing settings to inform self-identity (Rubinstein & Parmelee, 1992).

Consideration of AIP through an intersection of functional and emotional relationships provides a framework for our study, which recognizes AIP as a sociospatial phenomenon (Ahn, 2017; Wiles et al., 2012). Transformations to AIP precipitated by changes that the COVID-19 pandemic has had on physical, social, and technological environments may affect the “fit” between people and places (Weil, 2021) and how people feel about places where they are aging. Pandemic-borne stressors have called attention to physical and social isolation that influences older people’s physical and mental well-being (Pietrabissa & Simpson, 2020). Changes that tangibly restrict opportunities for social life, decrease in-person interactions, and present obstacles to accessing services have been shown to negatively affect older people since the start of the pandemic (Lebrasseur et al., 2021). Public health safety measures have also been associated with increased sedentary behavior, contributing to declines in mobility, balance, and overall quality of life for older adults (Bailey et al., 2021). Enhanced economic risk and delayed medical treatment compound pressures that additionally affect the well-being of older people (Miller, 2020). Smart technologies such as sensors, apps, and medical monitoring devices are known to support AIP for many people, yet there remains a need to address gaps in digital access and literacy to help connect older people to online community services (Barbosa et al., 2019; Delello & McWhorter, 2017). Digital alternatives that have been put in place to mitigate the isolating impact of pandemic response measures (e.g., shelter-in-place orders) may be less beneficial to older adults who use the Internet less than younger persons (Martins van Jaarsveld, 2020).

Although research has been conducted on the medical and public health impact of COVID-19 on older people (Lithander et al., 2020), less is known about the social and ecological implications that rapid environmental change has on the lives and well-being of community-dwelling older adults (Finlay et al., 2022). To address this gap, we conducted a photovoice study that asked participants to characterize aging in the “right” place during the pandemic. Recognizing that where an older person lives must match their ability to age optimally and must meet their unique lifestyles and vulnerabilities (Golant, 2015), our research investigated older adults’ perceptions of the “right” set of supports needed to AIP in a positive way. Our findings highlight several characteristics that older people associate with meaning and support during circumstances of the pandemic response in the summer of 2021 when the Delta variant swept through the United States prompting social distancing restrictions in public places.

## Method

Photovoice (Wang & Burris, 1994, 1997) was the ideal method for this research as it involves participants taking photographs of meaningful places or objects, recording details about each place or object, and discussing photographs with researchers to identify salient issues and themes (Novak, 2010; Plunkett et al., 2013). This method has been used to elicit critical dialog about aging-related issues (Mahmood et al., 2012; Miller, 2020) and to understand environmental factors that support conceptualizations of healthy AIP (Chan et al., 2016; Kohon & Carder, 2014; Ronzi et al., 2020; van Hees et al., 2017). Photos can represent a range of experiences from everyday realities and perspectives and provide a medium to discuss and analyze the qualitative aspects of place; this allows researchers to understand myriad experiences and perspectives, and gain insight into opportunities that support social participation and engagement (Bai et al., 2020). The open-ended nature of questions utilized to prompt participants in photovoice is well suited to study the subjective and objective constructs of agency and belonging in the Integrative Framework of Person–Environment Exchange (Chaudhury & Oswald, 2019). In previous research, photovoice has shown how older people may identify supportive factors for aging that are different from those identified by caregivers and aging service providers (van Hees et al., 2017), underscoring the significance of bringing older peoples’ voices into research on places of aging.

### Participants

Participants in this study (*n* = 17) were recruited from Salt Lake County, UT, through a letter distributed electronically and in hard copy. Salt Lake County is the largest county in Utah, and approximately 10% of residents are over 65 years old (Salt Lake County Aging Services, 2018). Researchers used a university-maintained database of local aging service providers and a database maintained by the Osher Institute for Lifelong Learning to distribute recruitment materials. Institutional approvals were secured prior to the start of recruitment (IRB 00141945). Participation was voluntary, and participants could withdraw from the study any time. Inclusion criteria included being over the age of 65, living independently in the community, and being fluent in English. A $75 gift card was offered to participants who completed the study. Participants in this study lived in various housing types, including single-family dwellings and multistory apartment complexes; five participants lived alone, closely reflecting the current national average among adults over the age of 65 (Administration on Aging, 2020). Demographic data are summarized in Table 1. Personal identifiers were maintained in a confidential database, and participants were assigned a number (#1–17) to maintain anonymity. Only one participant withdrew from the study.

**Table 1.** Participant Data Summary

Summary	Participant data
Number of participants	17
Average age	74.5 years old Standard deviation = 3.6
Gender	14 female 3 male
Marital status	10 married 4 widowed 3 separated or divorced
Race	17 White (Caucasian)
Employment	2 employed part time 15 retired
Highest level of education (degree)	2 associate degree 8 college degree 7 graduate degree
Living alone/with another person	5 Alone 12 With Another Person
Residential dwelling type	13 Single-family home 2 Townhome 2 Multifamily apartment/condominium

### Data Collection

**Figure 1.**
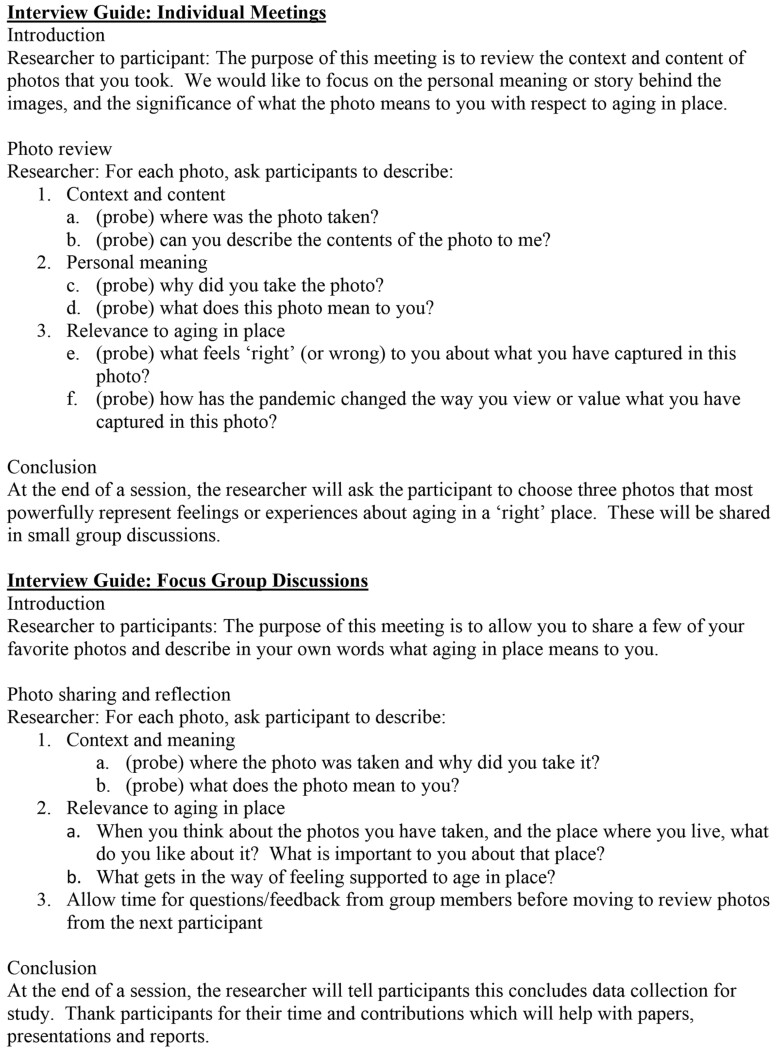
Interview questions: individual and group guides.

Researchers met individually with participants in an initial Zoom interview to collect basic demographic data and provide information on the goals and procedures of the study, including photo-taking practices and privacy protocols. Participants were asked to take a minimum of three photos per day for 1 week, and to keep a log of notes and photograph titles that captured their thoughts associated with each photo. A 1-week time period was identified to align with this study’s time and budget constraints. Researchers made disposable digital cameras available to participants as technology skills were not required for this study. However, all chose to use the camera functions on their personal cell phones or tablets. Participants electronically uploaded photos to a secure file-sharing location and met individually with research assistants for a 1-hr virtual interview to review and discuss their photo collections. At the end of each interview, researchers asked participants to identify their three favorite photos to share in a small group session after the individual interviews. Researchers facilitated three small group discussions on Zoom that allowed participants to present their photos and discuss meanings with fellow participants. Both individual interviews and group meetings were audio recorded, transcribed verbatim, and checked for accuracy by the research assistants. The approximate length of individual and group meetings was 1 hr. Table 2 summarizes our data collection steps. Figure 1 lists the questions researchers asked in individual and group sessions. Questions were designed to elicit information in three categories in both individual and group interviews: context and content; personal meaning; and relevance to AIP.

**Table 2.** Photovoice Data Collection Procedures

Research procedures	Activities	Format	Modality	Duration
Step 1: Initial interview	Semi structured interview: study information, demographic data collection, and consent procedures	One-on-one: researcher and participant	Zoom	30 min
Step 2: Orientation training	Presentation of photovoicing as a research method, instructions, question, and answer	Small groups: researchers and participants	Zoom	30 min
Step 3: Photo taking	Participants take three photos per day; option to title and record notes in photolog	Participants conduct this independently	In person	7 days
Step 4: Follow-up interview	Participants upload photos; researchers conduct semistructured interviews with screen sharing to review photos	One-on-one: researcher and participant	Zoom	60 min
Step 5: Focus group discussion	Participants identify three favorite photos and share with focus groups; researchers facilitate sharing through semistructured focus group discussions	Small groups: researchers and participants	Zoom	60 min

### Data Analysis

Researchers analyzed interview data using a thematic analysis procedure that followed a series of sequential steps (Braun & Clark, 2006). Familiarization with data was ensured through reading transcripts and listening to audio recordings. The faculty researchers and assistants inductively generated an initial set of codes based on topics participants chose to photograph and discuss. Two research assistants independently coded transcripts using NVivo12 software (QSR International, 2018), and the codes were reviewed by the research team. Codes were visually mapped using Miro, an online whiteboard, which enabled the research team to cross-read and organize/reorganize the data to support the definition and grouping of codes into themes. Photos associated with the data were integrated into the mapping, and provided a visual reference for data organization and review. Narrative data were initially analyzed and sorted according to different place types—including residential, social, recreational, vocational, and spiritual environments—which maps to distinct settings that are readily understood through photographs. Inductive coding led to a codebook that included topics that cross-cut place types, such as “Activities with friends,” “Adaptions for safe aging,” and “Seeking new experiences.” The processes of writing and reviewing definitions contributed to the iterative nature of discerning layers of meaning (Braun & Clark, 2006). This process led to the identification of codes that emphasize the constructs of agency and belonging, and using the Integrative Framework (Chaudhury & Oswald, 2019), we reorganized data into a new set of themes and categories as place types did not strictly map to larger themes. Data saturation was found during the analysis based on the repetition of themes and the overlap of characteristics within categories (Morse, 2015). The themes discussed in individual interviews were also identified in data collected during group discussions, offering further evidence of data saturation. Qualitative rigor was achieved through analyst triangulation as the research team discussed themes and coding structure until consensus was reached (Barusch et al., 2011; Creswell & Miller, 2000).

## Results

**Figure 2.**
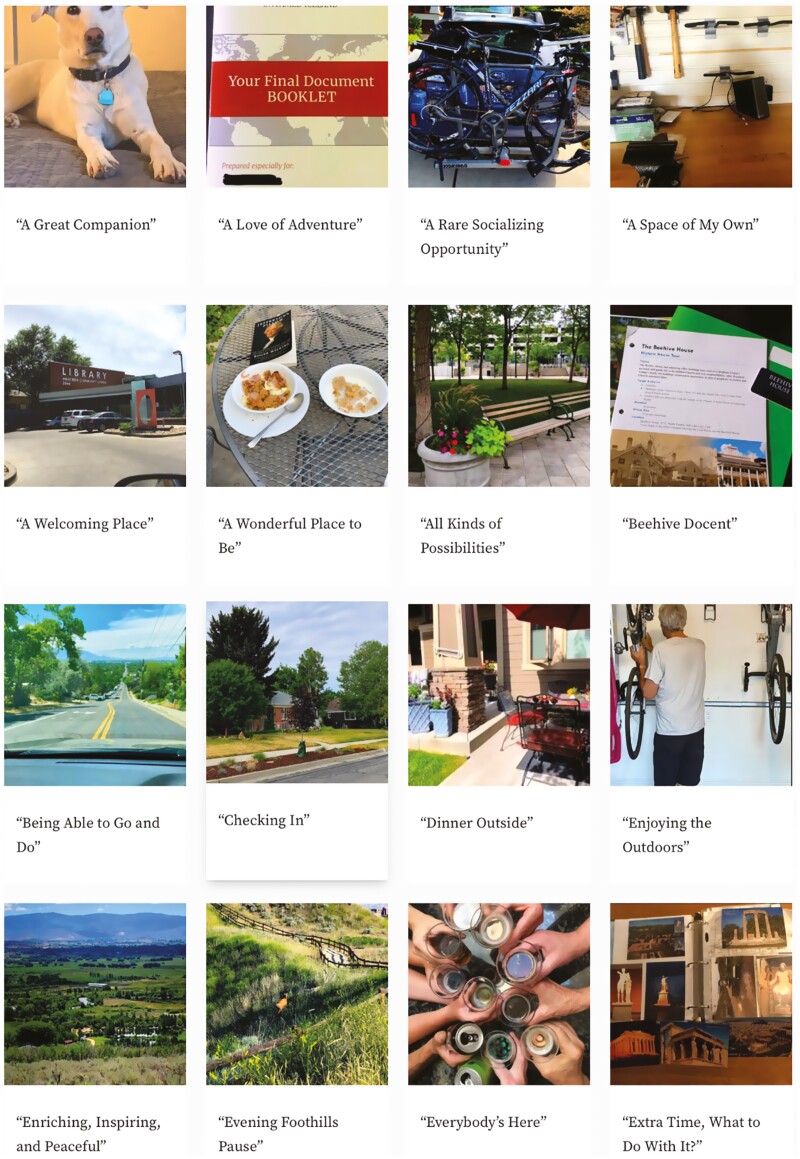
Sample of photos and captions generated by participants.

**Figure 3.**
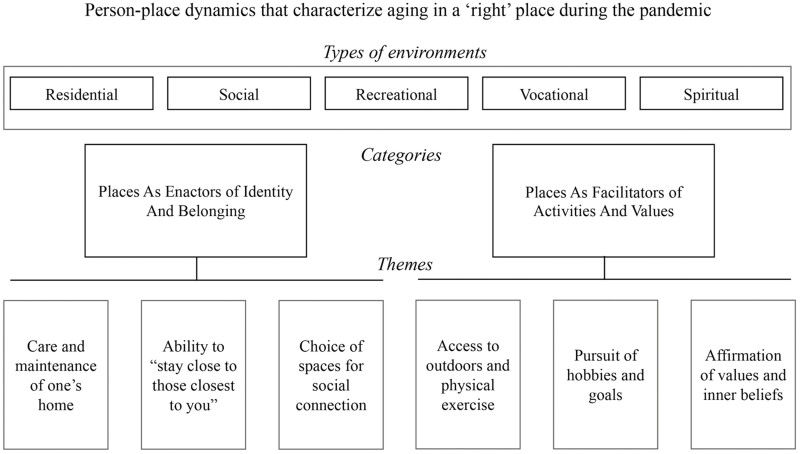
Characteristics of aging in a “right” place during the pandemic.

Six themes were identified and organized into two overarching categories. The first category, “Places as enactors of identity and belonging,” included caring for personal living spaces around the home, nurturing intimate relationships, and building social connections. The second category, “Places as facilitators of activities and values,” included exercising and being outdoors, pursuing goals and hobbies, and experiencing connection to values and belief systems. Approximately half of the photographs were taken in the interior of homes, reflecting residential and vocational domains where participants described their daily living environments and activities such as cooking, reading, computing, and gardening. The other half of the photographs were taken in spaces outside of participant’s homes, reflecting social and recreational domains such as neighborhood blocks, community spaces, and walking trails. A sample of photos and captions created by participants are shown in Figure 2. A conceptual model of the categories and themes that capture dimensions of aging in the “right” place during the pandemic is presented in Figure 3.

### Places as Enactors of Identity and Belonging

Participants characterized aging in a “right” place with ways that places support their own sense of identity and independence, as well as belonging and connection.

#### Care and maintenance of one’s home

Participants described caring for one’s own home as an expression of independence and identity; they spoke about how maintaining their homes engaged their hands, bodies, and minds and provided an outlet during the pandemic: “To feel useful or to feel creative; to be able to do repairs around the house feels good.” Describing a photo of a home maintenance project, a participant reflects:

In the pandemic, I think it was good for us … I’m not saying we did a whole lot more in terms of complexity than [we] would have done otherwise, but I think it was another added stimulus to stay involved and really be more involved. And I think there was a certain amount of pleasure taken in doing this … if you think about it in terms of keeping your environment around you healthy and, you know, positive as you get older.

Some participants emphasized the significance of places supporting how they take care of their homes, such as a workbench or supply area. In contrast, others described “pandemic projects” focusing on home improvements. Active engagement with home living environments contributed to a sense of purpose, identity, and independence in this theme.

#### Ability to “stay close to those closest to you”

Places that allowed participants to gather in-person with family and close loved ones contributed to participants’ sense of connection and belonging. Photographs associated with this theme were characterized by changes in that participants noted how they gathered with loved ones to maintain social distancing. One participant took a photo of her dining room table with eight place settings before she hosted her first dinner with her immediate family after everyone was vaccinated. She titled the photo “Anticipation,” saying, “I value this so much because we didn’t do it during the pandemic … it’s the small groups of people, the people I care most about getting to be together in person.” Another participant photographed her backyard patio, pointing out, “Six chairs at the table, so when it’s our immediate family, we can all sit there and we’re there a lot … we could all sit outside and have dinner together, and we weren’t uncomfortable about that.” Other participants noted sharing more time with grandchildren during the pandemic, filling the need for childcare. As one participant relayed, “[My grandsons] spent a lot of time with me because their parents both stayed at work. I got to know them a lot better … They’re just as happy at my house as at home.” Another participant photographed the sidewalk of her street, which was significant because her daughters lived a few blocks away; she says, “This matters, I think, in the broad sense of the pandemic because … older people want to be near family. I never really got it, that it was as important as it is.” The significance of place in this theme is closely associated with family connections and the ability to sustain in-person contact.

#### Choice of spaces for social connection

Participants also described the importance of places that allowed them to stay connected with friends. Neighborhood settings were places often associated with maintaining social connections. For instance, a participant took a photo of her block and said, “One of the things that I really like where I live is there are at least four or five single women in my age group on my two-block street. And during the pandemic, we were hollering across the street together, and that was great.” Other participants photographed community garden spaces, which allowed them to stay connected with neighbors and casual acquaintances while also being in a group setting in outdoor locations. Findings in this theme were also characterized by digital platforms utilized in response to the pandemic. For instance, participants described sharing interests in hobbies with friends and using online platforms such as Zoom and FaceTime. One participant described her relationship with a friend who shares her interest in quilting saying, “Even though we haven’t seen each other much over the last year, we exchange pictures constantly; we do Zoom calls, we talk about what projects are coming up and what our next trip will be.”

### Places as Facilitators for Activities and Values

Participants also associated a sense of aging in a “right” place with ways that places hosted meaningful activities and values.

#### Access to the outdoors and physical exercise

Nearly all participants described the significance of places that provide opportunities to be physically active and outdoors as a vital component to aging in a “right” place. Locations, where participants chose to exercise, were identified both inside and outside their homes. Participants shared photos of how they adapted spaces in their garages or living rooms to accommodate exercise equipment. They also cited the cancelation of group exercise classes in locations such as senior centers as reasons they adapted their exercise routines. Several participants joined Zoom-based exercise classes to support their daily routines. Participants also took photos of paths, sidewalks, and trails they frequented as places that enabled physical distancing while getting out and exercising. Integral to how participants described staying physically active was the feature of companionship in being outdoors. One participant took a photograph of bikes loaded onto the back of her car, saying

[My husband and I] biked in different places all within an hour, hour-and-a-half, of Salt Lake City, and with our pod, a couple, the other couple that we did this with, that was practically our only contact with people except for our immediate family.

Regular physical activity was also commonly achieved with dog walking, another form of companionship associated with positive AIP experiences: “I really value that we have a place like Mile High (Canyon), where we can go with our dogs off-leash and just walk back and forth … we were up there a lot during Covid.” These examples demonstrate how participants commonly associated companionship with physical exercise and outdoor spaces.

#### Pursuit of hobbies and goals

Participants described places dedicated to learning and pursuing goals as an essential component to aging in a “right” place. As one participant explained, “What our house allows us to do is very important … I’m a fabric artist, and I sew and I paint, and having the space to be able to do that in my own house is really lovely.” Spaces adapted to accommodate technology during the pandemic, such as computers, video cameras, and printers, were frequently described by participants as a kind of “central station” for daily activities, particularly for those who engaged in online learning or part-time work. Participants also described the importance of reading and photographed spaces where they like to read. One participant photographed journals filled with handwritten notes from a self-study of Russian, Latin, and Spanish languages, whereas another participant described reading thick history books. Participants described the significance of public libraries offering curbside delivery to check out books in their intellectual pursuits. Stacks of books and magazines and screens of laptops and computers were photographed and described by one participant as “really helpful to aging in place because having some work to do helps you feel relevant.” Various interior, exterior, and digital settings facilitated activities and goals that supported a sense of aging in a “right” place.

Exploration and discovery were strong components within this theme. The ability to get out of the house was a goal described by nearly all participants in this theme albeit in limited ways due to the pandemic. Participants described urban and rural destinations that were significant to them, mainly in outdoor settings, and underscored the importance of diversity among places. “The variety of topography, the variety of vegetation, the variety of experiences we’re able to have … is important to aging,” reflects a participant in describing a photo she had taken of the natural spaces near her home. Transportation was considered an integral component of AIP as participants described using their cars to get around town.

Salt Lake was locked down pretty good. We didn’t go out a lot. We’d go in our car and would drive … sometimes, just getting out to go to the grocery store was like, ‘I got to get out of this house’. Aging in place, you need some way of transportation, I mean otherwise, you can’t go to different places.

These descriptions indicate how community-based places and home-based settings facilitate activities that provide outlets, and how transportation is significant to access these sources of fulfillment outside the home.

#### Affirmation of values and beliefs

Meanings that participants assign to their daily environments further contribute to a sense of aging in a “right” place. Different aspects of place served as visual reminders of personal values and beliefs. For instance, a participant took a photo of a sidewalk near her house where she likes to walk, describing how it is significant because:

Every time I go [to this neighborhood], I’ll see a sign like Black Lives Matter or lots of Pride signs, colorful signs … It gets me out of the house because I know I can walk somewhere close and safe … when I walk this neighborhood, I see just hope and delight.

Other participants photographed places in their homes set up for religious worship or spiritual retreat, ranging from Catholic faith to Buddhist practice. One participant notes the significance of this during the pandemic:

I’m not a true Buddhist but I like their philosophies about life and death and transition, and constant change … it really helps me as I age … [the Buddha statue] just reminds me to laugh and smile when things get weird, and [during the pandemic] they were pretty weird.

Streams mountains, and lakes were photographed and described as settings that inspired a sense of being part of a world larger than oneself. Several participants photographed cloud formations and sunsets. These visual manifestations prompted participants to describe life perspectives that helped them contextualize stress and anxiety. Meanings that participants assign to their environments point to ways places helped inspire affirmation of values and belief systems among participants, reinforcing a positive perception of aging in a “right” place during the pandemic.

## Discussion

This study asked community-dwelling older adults to describe characteristics of where they live which support perceptions of aging in a “right” place during the pandemic. While AIP involves supporting older people to live as long as possible in their homes and communities, aging in the “right” place recognizes that where an older person lives impacts their ability to age optimally and must match their unique lifestyles and vulnerabilities (Golant, 2015). Findings advance aging research by demonstrating that conceptualizations of AIP extend well beyond housing and home even amidst the public health crisis of the pandemic. Data collection took place during the surge of the COVID-19 Delta variant, yet participants took almost as many photographs in community domains as they did in their homes in order to identify personal conceptions of what the right place looks like to them. Additionally, the findings of this study advance a conceptualization of aging in a “right” place by underscoring the relationship between emotional connections to place and the constructs of agency and belonging (Chaudhury & Oswald, 2019). Participants shared experiences of adapting their everyday environments due to social and physical changes tied to the pandemic, and described a sense of “rightness” associated with places that facilitated a sense of agency and belonging which supported these processes.

Consistent with previous research on place attachment (e.g., Giuliani, 2003; Low & Altman, 1992; Rubinstein & Parmelee, 1992), our findings suggest that empirical, objective aspects of place are highly integrated with emotional, subjective aspects. Objective characteristics highlight the “fit” between people and place and include home configurations that allow people to sustain hobbies, goals, and exercise routines. Adaptions described by participants included greater use of technology, increased time outdoors, and home modifications to support activities and interests. Subjective or emotional characteristics highlight ways that place reinforces a sense of connection and belonging, including places that support social gatherings and personal interactions. Adaptions in this domain include changing expectations and finding new ways to stay socially and emotionally connected while maintaining public health guidelines. Overall, we found little association in our study between residential type (i.e., single-family or multifamily dwelling) and types of adaptions that participants made to maintain a sense of aging in a “right” place. Yet, our findings reinforce the notion that disruption in life can deeply affect place attachment in a holistic way (e.g., Smith & Cartlidge, 2011), and suggest that the pandemic might have caused a transformation of older people’s sense of place attachment well beyond their home environment (Cowden et al., 2021). This notion of “home beyond the house” is important for understanding how older adults create meanings of home in later life (Cloutier-Fisher & Harvey, 2009), and is consistent with previous literature on the diverse and contested nature of the meaning of home for older adults in the face of changing circumstances (Oswald & Wahl, 2005), demonstrating applicability beyond the pandemic situation.

Our results point to distinct types of settings that support different levels of intimacy and contribute to a sense of aging in a “right” place during the pandemic. Areas around the home were commonly adapted for in-person gatherings with small groups of people or immediate family and loved ones. In contrast, communal and virtual settings were significant to hosting physically distanced exchanges with friends and neighbors. Both types of settings played a critical role in participants’ ability to stay connected with people and nurture relationships. The significance our participants described of places that provide in-person engagement is supported by research showing that limited face-to-face interactions and fewer regular activities correlate with higher rates of loneliness among older adults (Frenkel-Yosef et al., 2020; Fullana et al., 2020). Intergenerational relationships with children and grandchildren were particularly important to study participants, highlighting the role of spaces around the home and community that encourage connection between older and younger people. This is significant in the context of literature which suggests that decreased contact between generations as people adapt to social distancing guidelines may contribute to increased ageism and polarization, which has been seen since the onset of the pandemic (Sutter et al., 2022).

“Right” places described by older adults in this study also reflect multiple identities of place that change over time and in response to instability (Cutchin, 2003; Massey, 2005). The notion of “home as a refuge, community as a resource” (Oswald et al., 2011; Wiles et al., 2012) was upended during the pandemic when so many community-based locations were closed for prolonged periods. In response to change, participants in this study described outdoor, public open spaces as “right” places for physical exercise and social participation. “Right” places to exercise were highly connected to safe ways to socialize outside the home (Ghram et al., 2021). Valuing choice of settings for physical exercise may be especially important during the pandemic when social distancing limits the conditions that allow people to have physical contact with the outside world. Data also highlight the importance of diverse types of settings that support physical and mental health.

Finally, our findings lend insight into the proactive and reactive dynamics that create everyday experiences of aging in a “right” place. Participants adapted areas around their homes to host technology, arts and crafts, in-home exercise, and part-time work, which was critical to staying engaged in meaningful activities during the pandemic. Participants’ intellectual curiosities and creativity represented a broad range of interests, reflecting the tremendous heterogeneity and diversity among older people (Morrow-Howell & Gonzales, 2020).

There are several limitations to this study. All participants had their basic needs for food and housing met. Thus, our findings may not represent the unique experiences of AIP for vulnerable subgroups of people, including low-income older adults or older people experiencing homelessness (Canham et al., 2022; Park et al., 2017). Personal cell phones and computers were used to take photos and meet with researchers via Zoom, reflecting participants’ digital access and literacy. Although recruitment strategies included letters, flyers, and emails distributed through a variety of aging organizations, ranging from public housing to university-run continuing education programs, it may be that older adults who participated had means and resources to support their engagement in the study. Additionally, participants were all English speakers and Caucasian, which does not account for cultural differences that shape power dynamics and relationships to place (Mitra & Weil, 2016). Data captured through photovoice primarily represented micro and meso scales of place, but did not capture macro scales of place which are known to play a formative role in AIP (Hou & Cao, 2021). Nonetheless, studying AIP through photovoice methods that prioritize older people’s lived experiences provides important information with the capacity to positively inform the design, planning, and policy that will facilitate successful AIP (Weil, 2021). Future research should endeavor to replicate this study with minoritized populations in aging studies to capture macro-level influences on perceptions of AIP. Moreover, future research should address participation disparities in aging research by working to overcome barriers such as linguistic requirements, logistical issues, and access to digital technology (Gilmore-Bykovskyi et al., 2022). We recognize the critical need to invite culturally and ethnically diverse perspectives on how geographic locations affect aging.

## Conclusion

This article calls attention to the multiple aspects of people–place dynamics salient to aging in the “right” place within the crucible of the COVID-19 pandemic. Findings contribute to a growing body of literature that engages older adults’ voices and viewpoints to better understand the interactions between people and places that support the needs and goals that have been shaped by the global health crisis. The entwinement between physical and social meanings of place, as understood through lived experiences inside and outside of homes, compels a holistic view of places for aging. While the pandemic’s medical and public health impact on the aging population has been widely investigated, more attention is needed on how physical and social environments shape older adults’ quality of life and may inform positive experiences of AIP. Environmental transformations that impact aging in the “right” place will likely endure well beyond the pandemic, such as new uses of technology and new modalities for accessing resources. Lived experiences of older people provide essential data for policy-makers, caregivers, designers, and researchers. Given the relative recency of the pandemic, coupled with projections for rapid population aging and indication that most older people wish to AIP, further research is needed, particularly with diverse demographics of older people.

## Data Availability

The study reported in this manuscript is registered with the University of Utah’s Institutional Review Board (IRB 00141945). The study interview agenda can be requested from the corresponding author; no other study materials or data are available.
